# Registry of Stroke in Korean Medicine Hospital (RoS-KoMH): Protocol for a Prospective, Multicenter, Observational Study

**DOI:** 10.2196/67850

**Published:** 2025-11-25

**Authors:** Han-Gyul Lee, Woo-Sang Jung, Seungwon Kwon, Sangkwan Lee, Cheol-Hyun Kim, Dong-jun Choi, In Lee, Sang-Kwan Moon

**Affiliations:** 1 Kyung Hee University College of Korean Medicine Kyung Hee University Medical Center Seoul Republic of Korea; 2 Stroke Korean Medicine Research Center Wonkwang University Jeollabuk-do Republic of Korea; 3 Department of Korean Internal Medicine Dongguk University Ilsan Oriental Hospital Gyeonggi-do Republic of Korea; 4 Department of Internal Medicine of Korean Medicine Korean Medicine Hospital Pusan National University Gyeongsangnam-do Republic of Korea

**Keywords:** stroke registry, Korean medicine, quality improvement, Korean population, rationale and design, acupuncture, herbal medicine

## Abstract

**Background:**

Currently, conventional medicine for stroke treatment remains at a standstill. Korean medicine (KM), which is in high demand in Korea, has been shown to have a therapeutic effect on stroke; however, considering the absence of epidemiological and clinical characteristics of patients receiving KM for stroke in Korea and prospective, large-scale, long-term studies on the efficacy and safety of KM, the acquisition of data from KM treatments on stroke is essential.

**Objective:**

We aimed to collect and analyze data on the major clinical characteristics of patients with stroke receiving KM treatment and investigate the effectiveness and safety of KM in the Korean population.

**Methods:**

The Registry of Stroke in Korean Medicine Hospital (RoS-KoMH) is a prospective, multicenter, observational disease registry aimed at collecting data from 500 sets of patients at multiple timepoints. Eligible adult patients diagnosed with cerebral infarction, cerebral hemorrhage, or subarachnoid hemorrhage who visited 4 KM hospitals as outpatients or inpatients will be continuously registered using the electronic case report form. Baseline data at the first visit; KM treatment, rehabilitation therapy, and concomitant therapy during the visit; stroke evaluation every 4 weeks after the first visit; laboratory findings at discharge or the last visit; and safety evaluation information after each acupuncture or pharmacopuncture treatment will be collected.

**Results:**

The study was funded on August 19, 2020, by the Ministry of Health and Welfare, Republic of Korea, and recruitment for the study started on November 3, 2021. As of September 25, 2024, a total of 410 participants have been recruited.

**Conclusions:**

The RoS-KoMH study is the first and largest multicenter, prospective registry to record comprehensive data on KM treatment of stroke. The results of this study will provide high-quality evidence on the current state of stroke treatment using KM in actual clinical practice, as well as treatment effectiveness and safety, and will consequently contribute to the promotion and standardization of therapeutic interventions for stroke in Korea.

**Trial Registration:**

Clinical Research Information Service KCT0008494; https://cris.nih.go.kr/cris/search/detailSearch.do?seq=23999&search_page=L&search_lang=&class_yn=

**International Registered Report Identifier (IRRID):**

DERR1-10.2196/67850

## Introduction

Stroke is the second leading cause of death worldwide and is characterized by high mortality, morbidity, disability, and recurrence [1]. Although the mortality rate is decreasing owing to advances in risk factor control, treatment techniques (eg, thrombolysis), and secondary prevention, the incidence is gradually increasing owing to the rapidly aging population [2-4]. In addition, many survivors experience sequelae, and poststroke disability places a significant burden on patients, caregivers, and society [5].

Current treatments for stroke have made significant progress but still have distinct limitations. Thrombolysis is the recommended first-line treatment for acute ischemic stroke to improve long-term functional outcomes. However, access is limited by narrow time windows, high technical requirements, imaging dependence, and high costs [6]. Antiplatelets are recommended for most patients for the secondary prevention of ischemic stroke [7], but they have a recurrence rate of approximately 10%, have remained flat for decades [8], and carry a high risk of adverse effects, including a bleeding tendency [9]. For cerebral hemorrhage, there are no special treatment options other than blood pressure management unless surgery is indicated [10].

Korean medicine (KM), which includes acupuncture and herbal medicine, is a traditional medicine that falls under the category of traditional East Asian medicine and has a long history in Korea. More than 70% of Koreans experience KM treatment and report high treatment satisfaction [11]. As evidence accumulated on the effectiveness of KM treatment for stroke, the Korean Medicine Clinical Practice Guideline for Stroke was developed in 2021, recommending the use of KM treatments, such as acupuncture and herbal medicine, to improve symptoms, treat, and prevent stroke [12]. However, KM demand for stroke treatment is declining, with stroke as a reason for KM inpatient use decreasing from 20.4% in 2014 [13] to 3.3% in 2022 [11]. This is because the epidemiological and clinical characteristics of patients receiving KM for stroke have not been investigated in Korea, and KM for stroke has been criticized for its low-quality evidence of efficacy and safety. Therefore, there is a need for a large-scale, prospective study on KM in patients with stroke.

A registry is a method for building a unified system by recording and sharing large-scale data in the health care field. Ongoing stroke registries in Korea include the multicenter prospective stroke registry (Clinical Research Collaboration for Stroke in Korea) [14] and a multicenter, prospective cohort study on stroke rehabilitation (Korean Stroke Cohort for Functioning and Rehabilitation) [15]. However, these were both registry studies on Western medicine, and no registry studies on stroke involving KM have been conducted in Korea. In China, a multicenter registry of traditional Chinese medicine for stroke has been organized and is currently enrolling patients [16]. In Taiwan, the characteristics of usage and outcomes of traditional Chinese medicine treatments, such as acupuncture and herbal medicine, for stroke have been analyzed using large-scale, national health insurance data [17,18]. Therefore, there is an urgent need for a stroke registry study of KM in Korea.

We present the rationale and design of the Registry of Stroke at Korean Medicine Hospital (RoS-KoMH). The RoS-KoMH aims to establish a multicenter, prospective registry of stroke in KM hospitals to explore the demographics, clinical presentation, treatment usage status, therapeutic outcomes, and safety of patients treated in KM hospitals and to collect clinical evidence to improve future treatment outcomes.

## Methods

### Study Design

**Figure 1:**
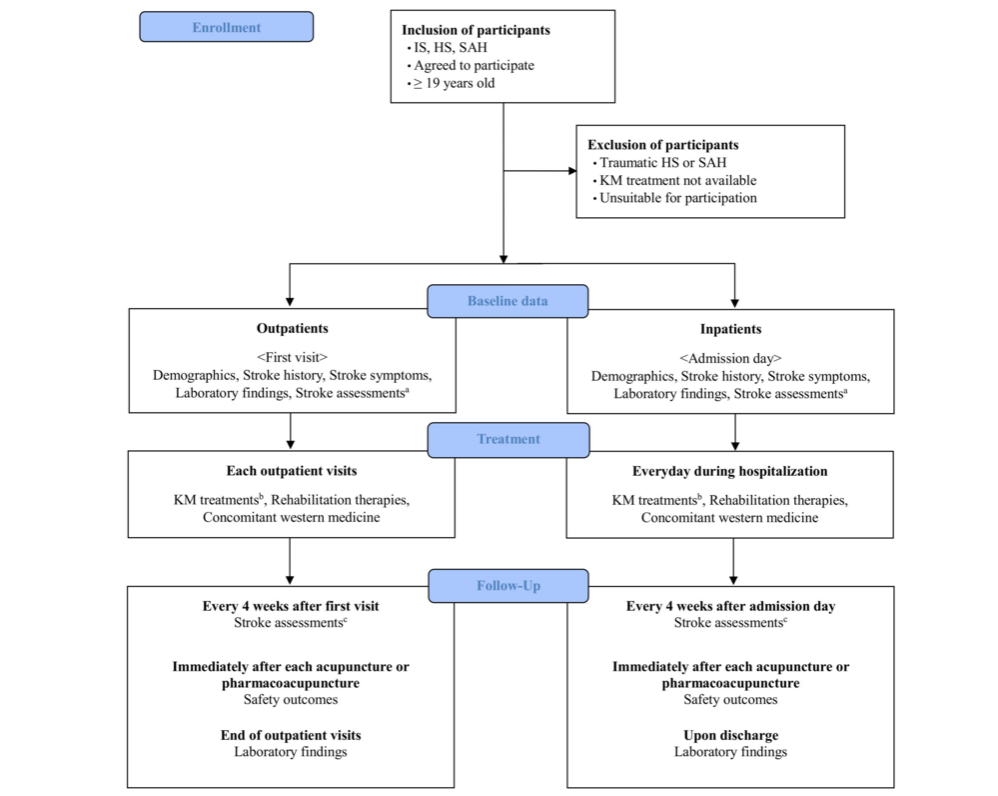
Flowchart of the study process. Stroke assessments at the initial assessment involve the manual muscle testing (MMT) grading system, modified Ashworth scale (MAS), grasp power, WIN-TRACK, timed up and go (TUG) test, modified Barthel index (MBI), fatigue assessment scale (FAS), fatigue severity scale (FSS), National Institutes of Health stroke scale (NHISS), Korean standard pattern identification (K-SPI) for stroke questionnaire, heart-kidney noninteraction questionnaire, Korean version of the mini-mental state examination (MMSE-K), and patient health questionnaire-9 (PHQ-9). Subsequent stroke assessments involve the MMT, MAS, grasp power, WIN-TRACK, TUG test, MBI, FAS, FSS, NHISS, K-SPI for stroke questionnaire, and heart-kidney noninteraction questionnaire. Korean medicine (KM) treatments include acupuncture, electroacupuncture, bee venom acupuncture, pharmacoacupuncture, indirect moxibustion, direct moxibustion, and cupping. HS, hemorrhagic stroke; IS, ischemic stroke; SAH, subarachnoid hemorrhage.

The RoS-KoMH is a voluntary multicenter, prospective, observational disease registry attempting to explore the demographics, clinical features, treatment status, therapeutic outcomes, and safety of patients with stroke in Korea who are treated using KM in KM hospitals and establish clinical evidence to improve treatment outcomes in the future. A flowchart of the study process is shown in Figure 1. This study will be conducted by the Kyung Hee University Korean Medicine Hospital (KH) located in Seoul, Dongguk University Ilsan Korean Medicine Hospital (DG) located in Gyeonggi-do, Wonkwang University Gwangju Medical Center (WK) located in Gwangju, and Pusan National University Korean Medicine Hospital (PS) located in Gyeongsangnam-do. The protocol was registered with the Clinical Research Information Service (KCT0008494) on June 2, 2023, and reported in accordance with the SPIRIT (Standard Protocol Items: Recommendations for Interventional Trials) checklist (Multimedia Appendix 1).

### Study Participants

#### Inclusion Criteria

The inclusion criteria for the study are as follows:

1. Men and women aged 19 years or older.
2. Patients diagnosed with a stroke (including ischemic stroke, hemorrhagic stroke, or subarachnoid hemorrhage; 10th revision of the International Classification of Diseases codes I60-I63) based on the results of clinical examination and radiologic examination and receiving treatment at an outpatient clinic or an inpatient ward of a KM hospital.
3. Patients who understood the purpose and intent of the study and voluntarily agreed to participate through written consent from the patient or their caregiver or representative.

#### Exclusion Criteria

The exclusion criteria for the study are as follows:

1. Patients with traumatic hemorrhagic stroke or traumatic subarachnoid hemorrhage.
2. Patients who suffer from diseases that can affect acupuncture, electroacupuncture, pharmacopuncture, bee venom injection, moxibustion, or cupping (eg, clinically severe skin diseases and blood clotting disorders) and therefore cannot be treated.
3. Patients judged by the investigator to be physically and mentally unsuitable for participation in the registration study based on examinations and laboratory findings.

#### Dropout Criteria

The following patients will be considered dropouts:

1. Patients discharged within 4 weeks of admission or lost to follow-up within 4 weeks of the first outpatient visit.
2. Participants who are noncompliant with the investigator’s instructions.
3. Participants who withdraw consent or request discontinuation.

### Number of Participants

The purpose of this study was not to statistically verify the intervention effect of KM treatment for stroke but to explore the demographics, clinical aspects, treatment status, and therapeutic efficacy and safety of KM for patients with stroke patients in Korea and to provide clinical evidence to improve the therapeutic effect of KM treatment in the future.

Accordingly, a total of 500 sets of clinical information on patients with stroke will be collected based on the opinions of the researchers, considering the financial resources, the environment in which the study is conducted, and the appropriate number needed to achieve the goal. One set was defined as a stroke patient for whom information was collected at the time of the first outpatient visit or admission, and additional information was collected at any point (4, 8, or 12 weeks) after the first outpatient visit or admission. For example, if information was collected for a single patient once upon admission, once in the fourth week of hospitalization, and once again in the eighth week of hospitalization, 2 sets of information would be considered collected.

### Recruitment of Participants

The participants of the study will be patients with stroke who visited as outpatients or were admitted as inpatients to KM hospitals. Patients who are deemed suitable for participation in the study based on the inclusion and exclusion criteria will be recruited by obtaining voluntary written consent from the participants or their caregivers within 7 days of their outpatient visit or hospitalization at a KM hospital after the research staff have provided all information related to the study. All information obtained from patients was collected and recorded using an electronic safety case report form (eCRF) that was developed and validated by the principal investigators and research staff from all institutions participating in the study (KH, DG, WK and PS) over a period of approximately 10 months through multiple face-to-face and web-based meetings, and all assessment tools included in the eCRF were developed, validated, and used in clinical practice.

### Baseline Data

Information on participant demographics (age, gender, type of health insurance, and smoking and drinking history), stroke history (stroke onset and initial diagnosis date; the type and date of computed tomography or magnetic resonance imaging used to diagnose the stroke; the site, type and lesion of the stroke; the Trial of Org 10172 in acute stroke treatment classification for cerebral infarction; history of stroke surgery; stroke risk factors; and past medical history), stroke symptoms (hemiplegia, sensory disorder, dysphagia, dysuria, dyspnea, digestive disorder, poststroke depression, poststroke delirium, poststroke insomnia, poststroke cognitive impairment, headache, dizziness, fatigue, and other symptoms), laboratory findings (complete blood count, biochemistry, lipid profile, and endocrine panel), and stroke assessment (hemiplegia with the manual muscle testing grading system, modified Ashworth scale, and grasp power; gait and balance disorders with the WIN-TRACK and timed up and go test; activities of daily living with the modified Barthel index; fatigue with the fatigue assessment scale and fatigue severity scale; stroke scales with the National Institutes of Stroke scale, Korean standard pattern identification for stroke questionnaire, and heart-kidney noninteraction questionnaire; impaired memory and disorientation with the Korean version of the mini-mental state examination; and depression with the patient health questionnaire-9) will be collected from medical records at the time of the first outpatient visit or inpatient admission. Detailed information regarding the collection of each item is presented in Table 1, and the participant timeline is presented in Table 2.

**Table 1:** Collecting information from participants.

Item	Information collected
Demographics	Age, gender, type of health insurance (national health insurance, medical benefits, or other), smoking history (status, past and present smoking, and amount of smoking), drinking history (status, past and present drinking, amount of drinking by type, and duration of drinking).
Stroke history	Stroke onset date and initial diagnosis date, the type and date of the imaging test used to diagnose the stroke (CT^a^ or MRI^b^), the site of the stroke (cortical area or subcortical area), the type of stroke (cerebral infraction, cerebral hemorrhage, or subarachnoid hemorrhage), the lesion of the stroke (ACI^c^, LACI^d^, or POCI^e^), TOAST^f^ classification for cerebral infarction (LAA^g^, CE^h^, SVO^i^, other causes, or undetermined), history of stroke surgery, stroke risk factors (previous stroke, hypertension, dyslipidemia, diabetes mellitus, atrial fibrillation, or others), and past medical history (date of diagnosis or surgery, name of disease or surgery, and current status).
Stroke symptoms	Hemiplegia (motor disorder), sensory disorder (including central pain), dysphagia (applying L-tube, PEG^j^, or dysphagia diet), dysuria (applying foley catheter), dyspnea (applying tracheostomy tube or oxygen), digestive disorder (constipation, dyspepsia, diarrhea, or others), poststroke depression (taking antidepressant or suspected depression on a depression-related scale), poststroke delirium, poststroke insomnia, poststroke cognitive impairment (taking drugs related to cognitive impairment or suspected cognitive impairment on a scale such as MMSE-K^k^), headache, dizziness, fatigue, and other symptoms.
Laboratory findings	Complete blood count (white blood cell; red blood cell; hemoglobin; hematocrit; platelet; differential count, including segmented neutrophils, lymphocytes, monocytes, eosinophils, and basophils; PT-INR^l^; and aPTT^m^), biochemistry (total bilirubin, protein, albumin, AST^n^, ALT^o^, BUN^p^, creatinine, sodium, potassium, chloride, calcium, uric acid, and glucose) , lipid profile (total cholesterol, triglyceride, HDL^q^-cholesterol and LDL^r^-cholesterol), and endocrine panel (hemoglobin A1C).
Stroke assessments	Hemiplegia with the MMT^a^, MAS^t^, and grasp power; gait and balance disorders with WIN-TRACK^u^ and TUG^u^ test; ADL^v^ with MBI^w^ and fatigue with FAS^x^ and FSS^y^; stroke scale with the NIHSS^z^, K-SPI^aa^ stroke questionnaire, and heart-kidney noninteraction questionnaire; impaired memory and disorientation with the MMSE-K; and depression with the PHQ-9^ab^
Korean medicine treatments	Herbal medicine (prescription name, administration purpose and duration, current administration, frequency, single dose, and unit), acupuncture (method, frequency, and duration), electroacupuncture (stimulus method, frequency, and duration), bee venom acupuncture (type and method, frequency, and duration), pharmacopuncture (type and method, frequency, and duration), indirect moxibustion (method, frequency, and duration), direct moxibustion (method, frequency, and duration), and cupping (method, frequency, and duration)
Rehabilitation therapies	Physical therapy, occupational therapy, speech therapy, and swallowing therapy
Concomitant treatment	Western medicine (ingredient name, administration purpose and duration, current administration, frequency, single dose, unit, route of administration)
Safety outcomes	Spot bleeding (bleeding time <10 s), microbleeding 1 (bleeding time 10 s to <20 s), microbleeding 2 (bleeding time 20 s to <30 s), extensive bleeding (bleeding time ≥30 s), massive hemorrhage influencing vital signs or causing other complications, bruising (checked 1-3 hours after removing the needles), edema, faintness or dizziness, fatigue or exhaustion, nausea or vomiting, pneumonia, needle fracture, skin eruption or itching, pain after needling, and other adverse effects after each treatment

^a^CT: computed tomography.

^b^MRI: magnetic resonance imaging.

^c^ACI: anterior circulation infarct.

^d^LACI: lacunar infarct.

^e^POCI: posterior circulation infarct.

^f^TOAST: Trial of Org 10172 in Acute Stroke Treatment.

^g^LAA: large-artery atherosclerosis.

^h^CE: cardioembolism.

^i^SVO: small vessel occlusion.

^j^PEG: percutaneous endoscopic gastrostomy.

^k^MMSE-K: Mini-mental state examination, Korean version.

^l^PT-INR: prothrombin time-international normalized ratio.

^m^aPTT: activated partial thromboplastin time.

^n^AST: aspartate aminotransferase.

^o^ALT: alanine aminotransferase.

^p^BUN: blood urea nitrogen.

^q^HDL: high-density lipoprotein.

^r^LDL: low-density lipoprotein.

^s^MTT: manual muscle testing grading system.

^t^MAS: modified Ashworth scale.

^u^TUG: timed up and go.

^v^ADL: activities of daily living.

^w^MBI: modified Barthel index.

^x^FAS: fatigue assessment scale.

^y^FSS: fatigue severity scale.

^z^NIHSS: National Institutes of Stroke scale.

^aa^K-SPI: Korean standard pattern identification.

^ab^PHQ-9: patient health questionnaire-9.

**Table 2:** Participant timeline for the Registry of Stroke in Korean Medicine Hospital.

Item	Outpatient first visit or inpatient admission day	At each outpatient visit or every day during hospitalization	Every 4 weeks after the outpatient first visit or inpatient admission day	Immediately after each acupuncture or pharmacopuncture. therapy	Final outpatient visit or upon discharge
Demographics	●				
Stroke history	●				
Stroke symptoms	●				
Laboratory findings	●				●
Stroke assessments^a^	●		●		
Stroke assessments^b^	●				
Korean medicine treatments		●			
Rehabilitation therapies		●			
Concomitant treatment		●			
Safety outcomes				●	

^a^Hemiplegia with the manual muscle testing grading system, modified Ashworth scale, and grasp power; gait and balance disorders with WIN-TRACK and the timed up and go test; activities of daily living with the modified Barthel index; fatigue with the fatigue assessment scale and fatigue severity scale; stroke scale with the National Institutes of Stroke scale, Korean standard pattern identification-stroke questionnaire, and heart-kidney noninteraction questionnaire; impaired memory and disorientation with the mini-mental state examination, Korean version; and depression with the patient health questionnaire-9.

^b^Impaired memory and disorientation with the mini-mental state examination, Korean version, and depression with the patient health questionnaire-9.

### Korean Medicine Treatments

Information on the use of herbal medicines (including herbal decoctions, inpatient pharmaceuticals, and external pharmaceuticals), acupuncture, electroacupuncture, bee venom acupuncture, pharmacopuncture, indirect moxibustion, direct moxibustion, and cupping during outpatient visits and hospitalizations will be collected daily (ie, at each outpatient visit or daily during hospitalization). The start and end dates of each treatment tool will also be investigated. If the use of any treatment tool has been discontinued, the date on which it was discontinued shall be the primary end date, the date the treatment was resumed shall be the secondary start date, and the date the treatment was last used shall be the secondary end date. For herbal medicine and pharmacopuncture, the prescription name will be written separately in the comments section of the eCRF. For herbal medicines, the start and end dates of each prescription will be recorded whenever the prescription is changed.

### Rehabilitation Therapies

Information on the use of physical, occupational, speech, and swallowing therapies during outpatient visits and hospitalizations will be collected every day (ie, at each outpatient visit or daily during hospitalization). The duration of each treatment will be recorded in the same way as that for the KM treatment.

### Concomitant Treatment

We will also collect information on the daily use of western medications (oral and injection types) every day (ie, at each outpatient visit or daily during hospitalization). For each western medicine, the name of the ingredient, purpose of administration, start date, end date, frequency of administration, dose, unit, and route will be investigated. If the frequency, dose, unit, and route change for the same ingredient, information will be collected separately.

### Follow-Up Measures

Stroke assessments (outlined in Table 2) will be conducted every 4 weeks after the first visit for both outpatients and inpatients. Follow-up laboratory findings will be collected at the final outpatient visit or discharge.

### Safety Outcomes

During the treatment period, the safety of acupuncture treatments will be evaluated when the needles are removed immediately after each acupuncture, electroacupuncture, pharmacopuncture, or bee venom acupuncture treatment. Bleeding events with duration and complications, bruising, edema, faintness or dizziness, fatigue or exhaustion, nausea or vomiting, pneumonia, needle fracture, skin eruption or itching, pain after needling, or other adverse events after each treatment will be assessed. The detailed safety assessment items are listed in Table 1.

### Data Analysis

Statistical analyses will be performed with SPSS version 21.0 (IBM Corporation) by our biostatisticians at the RoS-KoMH. Descriptive statistics will be used to analyze the data, and all measures will be presented as means (SDs) for continuous variables and n (%) for dichotomous variables. For variables assessed more than once, the amount or rate of change from baseline to follow-up will be calculated.

### Ethical Considerations

The study protocol was approved by the Institutional Review Board of Kyung Hee University Korean Medicine Hospital (KOMCIRB 2021-06-003-001, approved on August 13, 2021), Dongguk University Ilsan Korean Medicine Hospital (DUIOH 2021-10-008-002, approved on August 30, 2022), Wonkwang University Gwangju Medical Center (WKIRB 2021/7-2, approved on September 29, 2021), and Pusan National University Korean Medicine Hospital (PNUKHIRB 2021-11-003-001, approved on December 22, 2021). This study is conducted in accordance with the principles of the Declaration of Helsinki. Participants who wish to participate will receive an explanation of the consent form and must provide written consent of their own free will. All data from participants will be simultaneously recorded in an eCRF with an identification code rather than the participant’s name to protect personal information.

## Results

The study was funded by the Ministry of Health and Welfare, Republic of Korea (grant RS-2020-KH088006) on August 19, 2020. Recruitment for the registry commenced on November 3, 2021, and is scheduled to end on December 31, 2026. As of September 25, 2024, a total of 410 sets have been collected: 171 from KH, 88 from DG, 125 from WK, and 26 from PS. At this point, no data analysis has been conducted.

## Discussion

With increasing interest in the effects, benefits, and adverse effects of various treatments, well-designed, large-scale registries related to the long-term clinical outcomes of patients with stroke should be prioritized by clinicians and researchers [19]. Here, we describe the rationale and design of a prospective, multicenter, registry-based observational study investigating the clinical characteristics, KM stroke management, and outcomes of patients with acute stroke in a real-world setting in Korea. This study is expected to provide comprehensive real-world data on the demographic characteristics, treatment patterns, clinical outcomes, and safety of KM in stroke care across multiple institutions in Korea.

Considering that the current evidence-based guidelines for acute treatment and rehabilitation for stroke still have limitations despite several advances over the last few decades, the need for more effective and safer treatments to improve the prognosis of patients with stroke is constantly increasing [16]. Clinical KM studies on stroke have shown the efficacy of combined herbal treatment for acute ischemic stroke [20], the secondary prevention effect of herbal extracts on ischemic stroke [21,22], the benefits of acupuncture for stroke rehabilitation [23], the prevention effect of acupuncture [24], the improvement of dysphagia following acupuncture [25], the effect of pharmacopuncture on poststroke shoulder pain [26], and the effect of herbal treatment on chronic subdural hemorrhage [27]. The safety of KM treatment for stroke has also been reported, including the safety of combining warfarin with herbal medicine [28], the safety of liver and kidney function with the coadministration of western medicine and herbal medicine in patients with stroke [29], and the safety of combining acupuncture with warfarin [30] and novel oral anticoagulants in patients with stroke [31]. Most of these were retrospective cohort studies, cross-sectional studies, or systematic reviews that evaluated specific treatments or effects, which are common research methods used to generate evidence for KM and have been criticized for the quality of evidence on efficacy and safety. Therefore, it is necessary to improve the quality of evidence for the use of KM for patients with stroke.

Some characteristics of KM have made it difficult to build high-level evidence for it. In other words, KM is an empirical medicine that has accumulated over a long period, and it is difficult to standardize and objectively evaluate treatment because it provides customized treatment according to patient characteristics and requires a long treatment period and long-term follow-up. One way to supplement these points is to collect and use large-scale data [32]. Large-scale data use studies on KM in Korea are rare. In Taiwan, 99% of people are enrolled in national health insurance, and most traditional medicine treatments, including traditional Chinese medicine, are covered by national health insurance and used to generate evidence and improve treatment quality [33]. However, in the case of health insurance databases in Korea, KM is limited in that data cannot be obtained because major treatments such as herbal medicine (with some exceptions), pharmacopuncture, and bee venom acupuncture are not covered by national health insurance. Therefore, we designed the RoS-KoMH to compensate for these limitations by collecting information that can be obtained when treating patients with stroke patients at KM hospitals that actually treat stroke.

The RoS-KoMH protocol has some limitations. Although the RoS-KoMH registry collects longitudinal and multidimensional data, multivariate analyses, such as regression modeling or adjustment for potential confounders, have not yet been applied, which may limit causal inference regarding treatment outcomes given the variability in patient comorbidities and interventions. In addition, since the RoS-KoMH is a real-world, hospital-based, observational registry, it relies on voluntary participation to reflect the natural flow of patients receiving KM treatment for stroke in a routine clinical setting, which may lead to selection bias. The results of this study will provide evidence on the efficacy and safety of KM treatment for stroke to facilitate and standardize the optimization of individualized interventions for stroke prevention and treatment in Korea. Therefore, it is suggested that a registry study focusing on KM treatment be conducted using a stratified or consecutive sampling design as a follow-up to this study.

## Appendix

Multimedia Appendix 1 SPIRIT checklist.

## Abbreviations

eCRF: electronic case report form
KM: Korean medicine
RoS-KoMH: Registry of Stroke in Korean Medicine Hospital
SPIRIT: Standard Protocol Items: Recommendations for Interventional Trials

## Data Availability

Data sharing does not apply to this protocol, as no datasets have been collected or analyzed.
